# Evolution of Rhizaria: new insights from phylogenomic analysis of uncultivated protists

**DOI:** 10.1186/1471-2148-10-377

**Published:** 2010-12-02

**Authors:** Fabien Burki, Alexander Kudryavtsev, Mikhail V Matz, Galina V Aglyamova, Simon Bulman, Mark Fiers, Patrick J Keeling, Jan Pawlowski

**Affiliations:** 1 Department of Botany, University of British Columbia, Vancouver, British Columbia, Canada; 2 Research Group Protozoology, Institute of Biology/Zoology, Free University of Berlin, Königin-Luise-Str. 1-3, D-14195 Berlin, Germany; 3 Department of Invertebrate Zoology, Faculty of Biology and Soil Science, St-Petersburg State University, Universitetskaja nab. 7/9 199034 St-Petersburg, Russia; 4 Integrative Biology Section, University of Texas at Austin, Austin, TX 78712, USA; 5 New Zealand Institute for Plant & Food Research Ltd, Private Bag 4704, Christchurch, New Zealand; 6 Department of Zoology and Animal Biology, University of Geneva, Geneva, Switzerland

## Abstract

**Background:**

Recent phylogenomic analyses have revolutionized our view of eukaryote evolution by revealing unexpected relationships between and within the eukaryotic supergroups. However, for several groups of uncultivable protists, only the ribosomal RNA genes and a handful of proteins are available, often leading to unresolved evolutionary relationships. A striking example concerns the supergroup Rhizaria, which comprises several groups of uncultivable free-living protists such as radiolarians, foraminiferans and gromiids, as well as the parasitic plasmodiophorids and haplosporids. Thus far, the relationships within this supergroup have been inferred almost exclusively from rRNA, actin, and polyubiquitin genes, and remain poorly resolved. To address this, we have generated large Expressed Sequence Tag (EST) datasets for 5 species of Rhizaria belonging to 3 important groups: Acantharea (*Astrolonche sp., Phyllostaurus sp.*), Phytomyxea (*Spongospora subterranea, Plasmodiophora brassicae*) and Gromiida (*Gromia sphaerica*).

**Results:**

167 genes were selected for phylogenetic analyses based on the representation of at least one rhizarian species for each gene. Concatenation of these genes produced a supermatrix composed of 36,735 amino acid positions, including 10 rhizarians, 9 stramenopiles, and 9 alveolates. Phylogenomic analyses of this large dataset revealed a strongly supported clade grouping Foraminifera and Acantharea. The position of this clade within Rhizaria was sensitive to the method employed and the taxon sampling: Maximum Likelihood (ML) and Bayesian analyses using empirical model of evolution favoured an early divergence, whereas the CAT model and ML analyses with fast-evolving sites or the foraminiferan species *Reticulomyxa filosa *removed suggested a derived position, closely related to *Gromia *and Phytomyxea. In contrast to what has been previously reported, our analyses also uncovered the presence of the rhizarian-specific polyubiquitin insertion in Acantharea. Finally, this work reveals another possible rhizarian signature in the 60S ribosomal protein L10a.

**Conclusions:**

Our study provides new insights into the evolution of Rhizaria based on phylogenomic analyses of ESTs from three groups of previously under-sampled protists. It was enabled through the application of a recently developed method of transcriptome analysis, requiring very small amount of starting material. Our study illustrates the potential of this method to elucidate the early evolution of eukaryotes by providing large amount of data for uncultivable free-living and parasitic protists.

## Background

Over the last few years, the phylogenomic approach was successful in untangling several aspects of the early evolution of eukaryotes. Most eukaryotic diversity has been assigned to one of few supergroups [1] and new relationships between these large assemblages have emerged. For example, the unexpected close evolutionary affinity of Rhizaria to two of the "chromalveolate" groups, stramenopiles and alveolates (the SAR group, or Harosa in [2]), was recovered in several phylogenomic analyses [3-6]. Even orphan lineages that have been very challenging to place within the eukaryotic tree, such as the telonemids and centrohelids, or the breviate amoebae, have recently been shown to be related to haptophytes and centrohelids or to Amoebozoa, respectively [7-9]. However, several question marks remain, notably concerning the placement of the root [10], the monophyly of some supergroups [11], and the relationships within and between the supergroups, especially where uncultivated protists dominate.

The supergroup Rhizaria, composed of several phyla that are difficult to maintain in laboratory cultures, is a good example of the persisting uncertainties for the relationships between the major members of this assemblage. Although a few rhizarians can be isolated and cultivated [12], the majority is known only from environmental sequences [13] or single-specimens extractions [14,15]. Consequently, rhizarians are represented in sequence databases almost entirely by ribosomal rDNA [16]. A few protein sequences of actin, α-tubulin, β-tubulin, RNA polymerase II, and polyubiquitin are available for selected taxonomic groups [17-20] but for other lineages, such as radiolarians, only the actin-coding gene has been sequenced, which is in sharp contrast to the great diversity of the group and its ecological importance. Recently, five small rhizarian cDNA libraries have been sequenced (3 Cercozoa and 2 Foraminifera), partially filling the gap in comparison to other supergroups, and one genome project (*Bigelowiella natans*) is in progress (http://www.jgi.doe.gov/sequencing/why/50026.html).

According to the current consensus, Rhizaria are composed of three highly diverse and possibly monophyletic phyla, Cercozoa, Foraminifera, and Radiolaria (including Acantharea, Polycystinea and Taxopodida, but excluding Phaeodarea that were shown to branch among Cercozoa [21]). The Rhizaria comprise also the parasitic Phytomyxea and Haplosporidia, as well as various marine filose and reticulose protists, including Gromiida and *Filoreta*, sometimes considered members of Cercozoa [22,23]. The relationships between these groups are uncertain, due to the lack of resolution observed in the SSU and LSU rDNA as well as the few available protein trees. The most controversial is the position of Foraminifera, whose fast evolving SSU rDNA sequences branch either close to Haplosporidia and Gromiida [19,24] or as sister group to Radiolaria [13,25,26]. The weakly supported grouping of Foraminifera and Radiolaria observed in some SSU and LSU rDNA trees led to the creation of the infrakingdom Retaria [26,27]. Another source of information came from the insertions of one or two amino acids at the monomer junctions in the highly conserved protein polyubiquitin. These insertions have been found in Cercozoa and Foraminifera but not in all other eukaryotes studied to date, including radiolarians [17,23]. It has been argued that the ancestor of polycystine and acantharean Radiolaria could have lost the insertion, but the lack of insertion could also be explained by contamination of DNA samples by non-rhizarian protists [23].

To test the Retaria hypothesis and to shed light on the relationships between most of the deeply branching rhizarian groups, a protocol was developed to prepare cDNA libraries suitable for 454 sequencing from a handful of cells collected from environmental samples. We obtained and analyzed more than 670,000 ESTs from 2 marine acantharean Radiolaria (*Astrolonche *sp. and *Phyllostaurus *sp.), 2 parasitic Phytomyxea (*Plasmodiophora brassicae *and *Spongospora subterranea*) and *Gromia sphaerica*, a giant marine testate protist that is capable of producing macroscopic bilaterian-like traces [28]. Phylogenetic analyses of 167 genes support the Retaria hypothesis and suggest that this group may be most closely related to Phytomyxea and *Gromia*. Moreover, our study confirms the presence of polyubiquitin insertion in some Acantharea and reveals another possible rhizarian-specific signature in one of the ribosomal proteins.

## Results

### Dataset construction

The phytomyxean *P. brassicae *and *S. subterranea *are parasites of the plant genera *Brassica *and *Solanum*, respectively, and the *in vitro *callus samples were prepared according to an unpublished protocol (Bulman et al. submitted). Consequently, an unknown amount of plant contamination was expected in the phytomyxean ESTs. An initial blast examination showed that many of the phytomyxid-callus contigs had high similarity to plant sequences and were thus possibly derived from the host cells. We took advantage of the large amount of data available for *Brassica *and *Solanum *to filter out these plant sequences and simplify data searching for constructing the single-gene alignments (see Methods).

A total of 167 gene alignments with at least one rhizarian species represented in each were constructed for phylogenetic analyses. Based on recently published results suggesting close evolutionary affinities between Rhizaria, stramenopiles and alveolates, forming the so-called SAR group [3,5,6], representatives for these 3 groups were included. The full dataset comprised 10 rhizarians, 9 stramenopiles, and 9 alveolates. In order to reduce the risks of artifacts, 11 green plant taxa were chosen to root our phylogenies because 1) of the availability of complete genomes for many lineages, thus considerably reducing the amount of missing data in the outgroup; 2) they have evolved more slowly comparatively to most of the SAR species; and 3) their relative evolutionary proximity to the SAR group in the tree of eukaryotes [4]. However, an alternative outgroup, haptophytes, was also tested as it was proposed to be more closely related to the SAR group (data not shown) [7]. We did not select it for the final analyses because only medium-sized EST datasets are available for a limited number of species, except for one complete genome (*Emiliana huxleyi*), and the intra-Rhizaria relationships remained identical to the trees rooted using the green plants (see below). Each single-gene dataset was thoroughly tested by bootstrapped maximum likelihood (ML) analyses for deep paralogy or suspicious relationships possibly indicative of lateral gene transfer (LGT) or contamination. The acanthareans are known to harbor zooxanthellae symbionts and polycystine radiolarians are hosts of prasinophytes, dinoflagellates and other alveolates [29]. Accordingly, non-acantharean sequences were expected to be found. Out of the 167 selected genes, we could identify 1 sequence related to haptophytes in *Astrolonche *and *Phyllostaurus*, 2 sequences of dinoflagellate origin in *Astrolonche*, 5 and 2 sequences of general plant affinity in *Astrolonche *and *Phyllostaurus*, respectively, and, surprisingly, 25 sequences in *Astrolonche *and 21 sequences in *Phyllostaurus *clearly belonging to streptophytes (angiosperms). It is not clear to us why streptophyte sequences were present in our acantharean dataset, but one possible explanation could be that the samples were contaminated with a small amount of pollen. All these contaminant sequences were removed from our alignments. The curated protein alignments were concatenated into a supermatrix amounting to 36,735 unambiguously aligned amino acid positions (global percentage of missing data: 40%; see Additional file 1 for details) that was subjected to phylogenetic analyses.

### Phylogenetic analyses of the supermatrix

**Figure 1:**
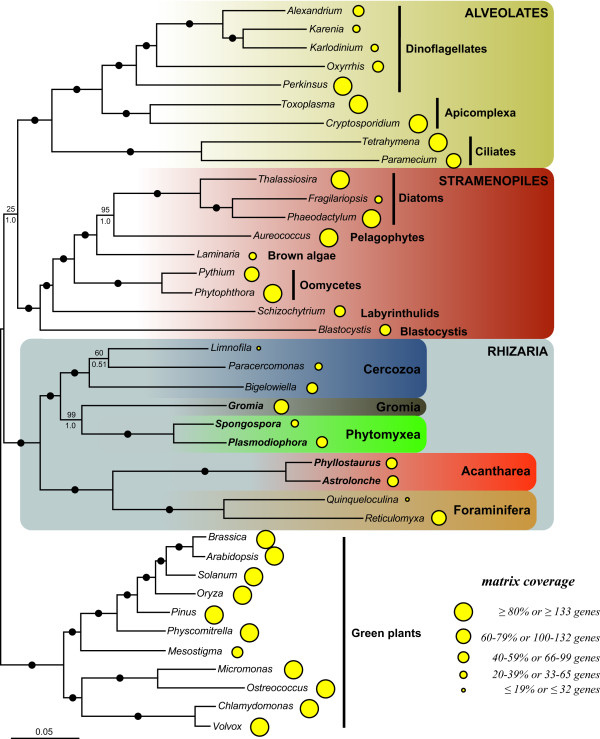
**Phylogeny of SAR as inferred by RAxML and the LG model of evolution**. The tree was rooted using the green plants. Species with new genomic data generated in this study are in bold. An identical topology was also recovered using MrBayes and the WAG model of evolution. Black dots correspond to 100% ML bootstrap support (BP) and 1.0 Bayesian posterior probabilities (PP). Numbers at nodes represent BP (above) and PP (below) when not maximal. The area of the yellow circles are proportional to the number of genes included in the supermatrix for each taxon. The scale bar represents the estimated number of amino acid substitutions per site.

**Figure 2:**
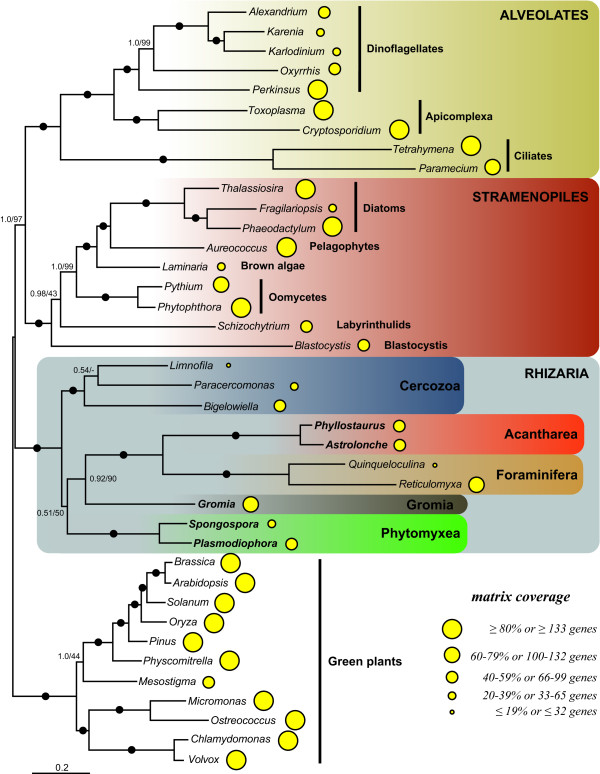
**Phylogeny of SAR as inferred by PhyloBayes and the CAT model of evolution**. Consensus tree between 2 independent Markov chains, rooted with green plants. Species with new genomic data generated in this study are in bold. Black dots correspond to 1.0 PP and 100% BP and values at nodes PP and BP when not maximal. The area of the yellow circles are proportional to the number of genes included in the supermatrix for each taxon. The scale bar represents the estimated number of amino acid substitutions per site.

We analyzed our concatenated alignment using probabilistic methods of tree reconstruction with (i) empirical site-homogeneous models of sequence evolution in ML (LG model) and Bayesian (WAG model) frameworks and (ii) site-heterogeneous mixture model in a Bayesian framework (CAT model). Figures 1 and 2 depict the relationships inferred from these analyses. The "LG" and "WAG" trees received maximal bootstrap support proportions (BP) and posterior probabilities (PP), respectively, for nearly all nodes (Figure 1). As expected, the 3 major groups included in this study, i.e. rhizarians, stramenopiles and alveolates, were strongly recovered, and the relationships between and within them corresponded to previously published trees [4,6,7]. All 3 analyses robustly supported the monophyly of the four rhizarian phyla: Foraminifera, Acantharea, Phytomyxea and Cercozoa. Notably, Foraminifera were placed as a sister group to Acantharea with 100% BP ("LG" and "CAT") and 1.0 PP ("WAG" and "CAT"). The branching order within Rhizaria was identical and highly supported in the "LG" and WAG" trees, with the Foraminifera + Acantharea clade being sister to Cercozoa and a group composed of Phytomyxea and *Gromia *(Figure 1). On the other hand, the site-heterogeneous CAT model inferred a different topology that received low to high PP and BP, recovering the Foraminifera + Acantharea group in an internal position, sister to *Gromia *(0.93 PP; 90% BP) and more closely related to Phytomyxea (0.51 PP; 50% BP) to the exclusion of Cercozoa (Figure 2). Because the discrepancies between the "LG" and "CAT" topologies are an indication that some relationships may be artifactual, we estimated the fit of these 2 models based on a cross-validation test (see Methods). The "CAT" model was found to much better fit the data than the "LG" model with a score averaged over 10 replicates of 1547 ± 71 (all replicates favoured the "CAT" model), indicating that the topology in Figure 1 is likely the results of biases not correctly handled by the site-homogeneous models (LG and WAG).

To better evaluate these differences, a topology comparison analysis using the approximately unbiased (AU) test was performed [30]. Both trees in figure 1 (*P *= 0.916) and in figure 2 (*P *= 0.084) were not rejected at the 5% significance level. This test was based on the comparison of trees obtained with 2 non-nested models, "LG" (Figure 1) and "CAT" (Figure 2), using the "LG" empirical matrix. Hence, if the topology in Figure 2 had been rejected, it would not have been very informative because the "CAT" model could still have inferred the true tree. In the present case, however, the LG-based AU test kept the "CAT" tree among the trees possibly correctly describing the relationships within Rhizaria, thus strengthening the branching pattern showed in Figure 2. In addition, a topology with Acantharea alone in a sister position to the rest of Rhizaria was also tested in order to estimate the likelihood of the basal branching of Radiolaria seen in some SSU trees (see [16] for a discussion). This topology was strongly rejected (*P *= 7e-09), further supporting the association of Foraminifera and Radiolaria.

### Evaluating the branching order within Rhizaria

**Figure 3:**
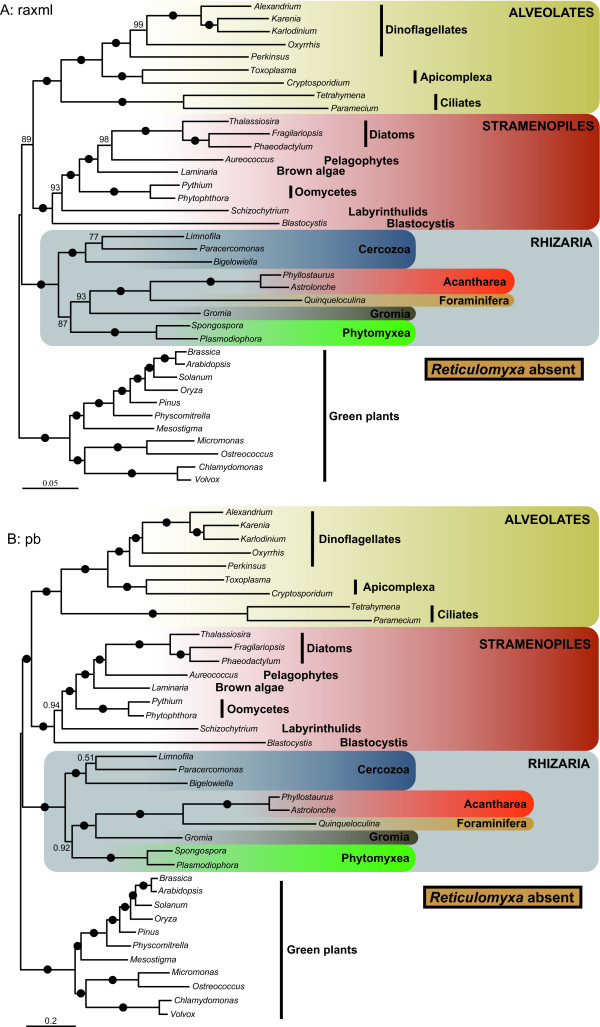
**Phylogeny of SAR without *Reticulomyxa***. RAxML with "LG" model (A) and PhyloBayes with "CAT" model (B) phylogenies of the SAR group, rooted with the green plants. The foraminiferan species *Reticulomyxa filosa *was removed from the alignment for inferring these trees. Black dots correspond to 100% ML bootstrap support (BP) in (A) and 1.0 Bayesian posterior probabilities (PP) in (B). Numbers at nodes represent BP (A) or PP (B) when not maximal. The scale bar represents the estimated number of amino acid substitutions per site.

**Figure 4:**
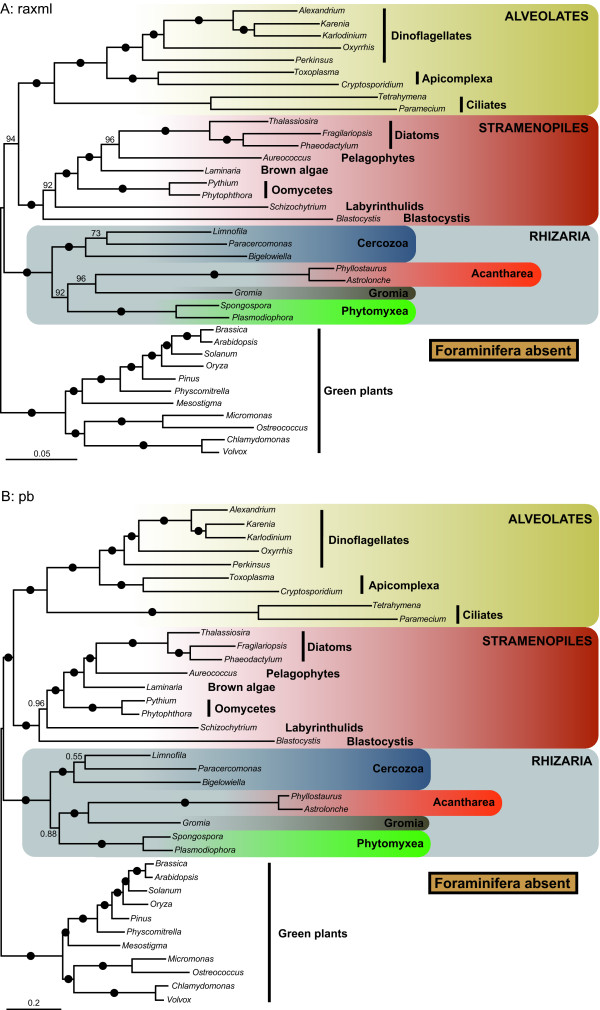
**Phylogeny of SAR without Foraminifera**. RAxML with "LG" model (A) and PhyloBayes with "CAT" model (B) phylogenies of the SAR group, rooted with the green plants. Both foraminiferan taxa *Reticulomyxa filosa *and *Quinqueloculina *sp. were removed from the alignment for inferring these trees. Black dots correspond to 100% ML bootstrap support (BP) in (A) and 1.0 Bayesian posterior probabilities (PP) in (B). Numbers at nodes represent BP (A) or PP (B) when not maximal. The scale bar represents the estimated number of amino acid substitutions per site.

**Figure 5:**
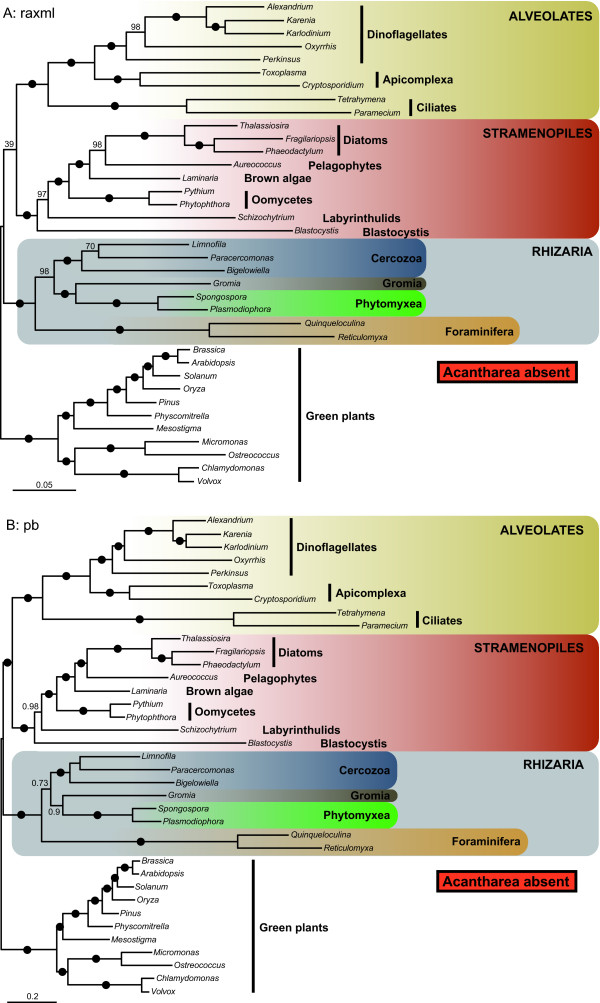
**Phylogeny of SAR without Acantharea**. RAxML with "LG" model (A) and PhyloBayes with "CAT" model (B) phylogenies of the SAR group, rooted with the green plants. Both acantharean taxa *Astrolonche *sp. and *Phyllostaurus *sp. were removed from the alignment for inferring these trees. Black dots correspond to 100% ML bootstrap support (BP) in (A) and 1.0 Bayesian posterior probabilities (PP) in (B). Numbers at nodes represent BP (A) or PP (B) when not maximal. The scale bar represents the estimated number of amino acid substitutions per site.

In our trees, both foraminiferans and acanthareans appeared as fast-evolving taxa. This raised a concern about their potentially erroneous grouping due to the long branch attraction (LBA) artifact [31] that would affect not only the position of these diverging lineages but also the relationships among all rhizarian groups. To evaluate for the possibility of LBA, we first conducted a fast-evolving taxa removal experience in which, in turn, the most diverging foraminiferan representative *Reticulomyxa filosa *(Figure 3), both foraminiferan species (Figure 4), and the acanthareans (Figure 5) were discarded. The removal of *R. filosa *had no impact on the sister relationship of foraminiferans and acanthareans: both groups remained monophyletic with maximum support. However, this slightly different taxon sampling largely affected the branching order among the rhizarian groups. The "LG" model robustly placed *Gromia *as the most closely related lineage to the Foraminifera + Acantharea group (93% BP), and Phytomyxea were recovered as sister to this assemblage with 87% BP (Figure 3A). The "CAT" model inferred the same topology (Figure 3B), which also corresponded to the full tree inferred with this model (Figure 2) but, interestingly, the support values increased from 0.92 to 1.0 PP and from 0.51 to 0.92 PP for the node joining Foraminifera + Acantharea + *Gromia *and the node uniting Phytomyxea to this group, respectively. Similarly, when Foraminifera were removed altogether, both models again recovered the "CAT" topology (Figure 2) with high BP and PP values, exactly as in absence of *Reticulomyxa *only (Figure 3). Finally, discarding Acantharea led in both "LG" and "CAT" analyses to the basal position of Foraminifera (98% BP; 0.73 PP) and the sister position of *Gromia *to Phytomyxea (100% BP; 0.9 PP), as observed in the complete "LG" tree (Figure 5 and Figure 1).

**Figure 6:**
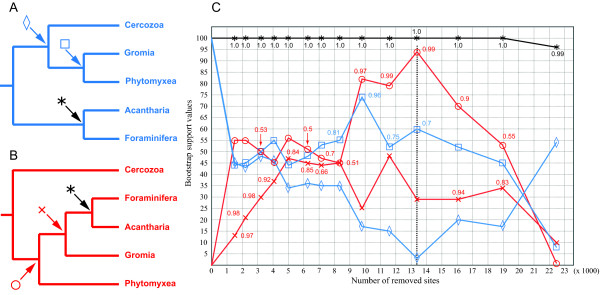
**Site removal analysis**. Figures (**A**) and (**B**) illustrate the bipartitions that were sought in the pool of trees generated by bootstrapped ML reconstructions, corresponding to the "LG" (blue) and "CAT" (red) relationships, respectively. The monitored relationships are indicated as followed: star: Foraminifera-Acantharea grouping; square: *Gromia *sister to Phytomyxea; diamond: basal position of the Foraminifera-Acantharea clade within Rhizaria; cross: *Gromia *sister to the Foraminifera-Acantharea clade; circle: basal position of Cercozoa to the rest of rhizarian lineages. (**C**) Dependence of the bootstrap support values (BP) for the monitored relationships on the number of removed fast-evolving sites, marked for each of the 14 shorter alignments. The blue and red lines correspond to the BP of nodes found in the "LG" and "CAT" trees, respectively. The black line corresponds to the BP for the Foraminifera-Acantharea grouping. The vertical dashed line shows the step (13'379 positions removed) where the supports for the sister position of Retaria reached a minimum and the support for the sister position of Cercozoa a maximum. Numbers next to the marks are PP obtained with the "CAT" model (PhyloBayes), resulting from the pooling of all trees after burnin of 2 independent chains and corresponding to the bifurcations found. At removal steps 1, 2, 4 and 5 only one PP value is shown next to the cross mark, indicating that the CAT model could not infer the position of Phytomyxea within Rhizaria (multifurcation). The y-axis represents the BP and the x-axis the length of the alignments after the removal of sites.

To assess the robustness of the Foraminifera-Acantharea clade and to further investigate the two competing topologies for intra-Rhizaria relationships (Figure 1 and 2), we then conducted a site removal analysis in which the fastest-evolving sites were progressively removed from the original alignment. The rationale behind this analysis is that fast-evolving sites are more likely to be saturated and not correctly interpreted as convergence by tree reconstruction methods, thus strongly influencing the potential artifactual grouping of highly diverging lineages [32]. Specifically, we tested 14 shorter alignments ranging from 35,230 aa to 14,281 aa and reconstructed phylogenetic trees with LG and CAT models at each step to determine the support value for several nodes of interest (Figure 6). First, the highly supported association between Foraminifera and Acantharea was not affected by the removal of fast-evolving sites, with almost no decrease in bootstrap values even for the smallest number of positions remaining in the alignment. This result provides additional evidence that the grouping of Foraminifera and Acantharea is not caused by artifacts of tree reconstruction. Second, we monitored the bootstrap supports for the sister position of *Gromia *with respect to Phytomyxea, the basal position of the Foraminifera-Acantharea clade (as observed in the "LG" tree, Figure 1), as well as the alternatives: the sister grouping of *Gromia *to the Foraminifera-Acantharea group, and the basal position of Cercozoa (as observed in the "CAT" tree, Figure 2). Interestingly, as the fast-evolving sites were removed, the bootstrap values for the phylogenetic relationships obtained in the LG-based analysis of the complete dataset decreased (Figure 6, blue line) and, at the same time, the branching order supported by the CAT-based reconstruction gained statistical significance (Figure 6, red line). When 13'379 fast-evolving positions were removed, the LG-based analysis converged with high support (94% BP; 0.99 PP) towards the topology that was weakly suggested by the CAT-based analysis of the complete dataset for the association of Phytomyxea, *Gromia*, Foraminifera and Acantharea, before diverging likely due to lack of phylogenetic signal in the shortest alignments. The position of *Gromia *remained more ambiguous throughout the removal process and, although the support for the association with Phytomyxea rapidly decreased to below 50% BP, its sisterhood to the Foraminifera-Acantharea clade suggested by the "CAT" model did not gain significance.

### Actin phylogeny

**Figure 7:**
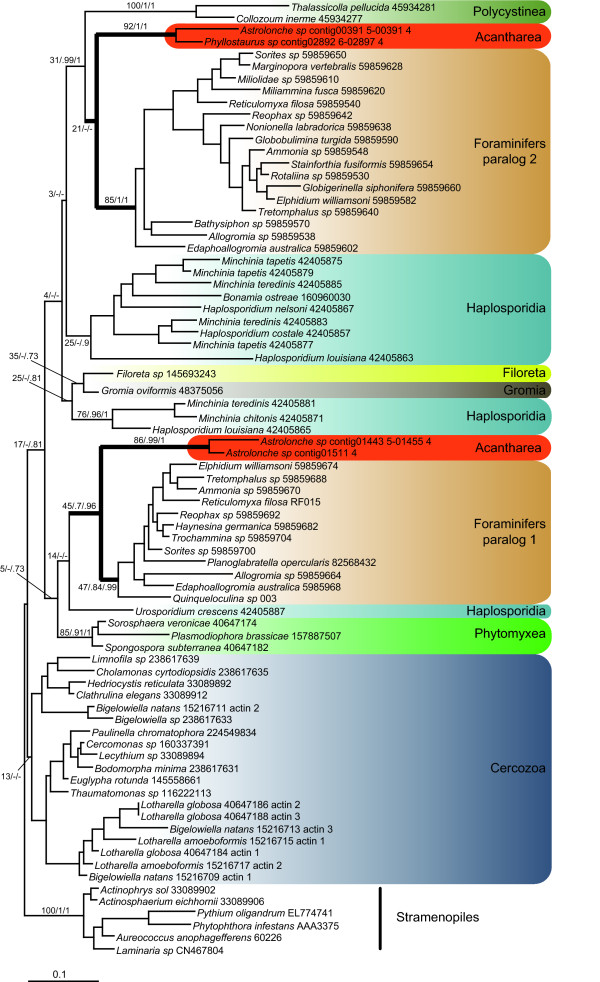
**Actin phylogeny of Rhizaria**. ML phylogeny of Rhizaria based on actin, rooted with stramenopiles as outgroup. Numbers at nodes represent the bootstrap values obtained with RAxML ("LG" model) and the posterior probabilities obtained with PhyloBayes ("LG" model) and MrBayes ("WAG" model). For clarity, only the values for the deep nodes and the nodes of interest for this study are shown The scale bar represents the estimated number of amino acid substitutions per site. The branches leading to Acantharea and Foraminifera in actin paralogs 1 and 2 are in bold.

Although our multigene analysis represents the broadest rhizarian sampling to date, three important rhizarian groups, Haplosporidia, *Filoreta*, and Polycystinea, are still missing. Therefore, we performed a separate phylogenetic analysis based on actin, the only protein-coding gene sequenced in all rhizarian groups. ML and Bayesian analyses of our alignment (317 amino acid positions), containing 73 rhizarians and 6 stramenopiles as outgroup, indicated that the acantharean *Astrolonche *possesses 2 actin paralogues branching as sister groups to 2 of the actin paralogues present in Foraminifera (Figure 7). The only actin sequence found in *Phyllostaurus *grouped with *Astrolonche *as sister to the foraminiferal paralogue 2. Sister to this clade were two previously obtained actin sequences of the polycystinean radiolarians *Thalassicolla pellucida *and *Collozoum inerme*, and their grouping with Acantharea and Foraminifera was strongly supported in Bayesian inferences (0.99 and 1.0 with PhyloBayes and MrBayes, respectively) but not supported in ML (31% BP). However, the relationships between these 3 groups remained unresolved, leaving open the question of a possible radiolarian monophyly. For both paralogues, Haplosporidia appeared as sister to the Foraminifera + Radiolaria clade, albeit without much support.

### Rhizarian signatures

**Figure 8:**
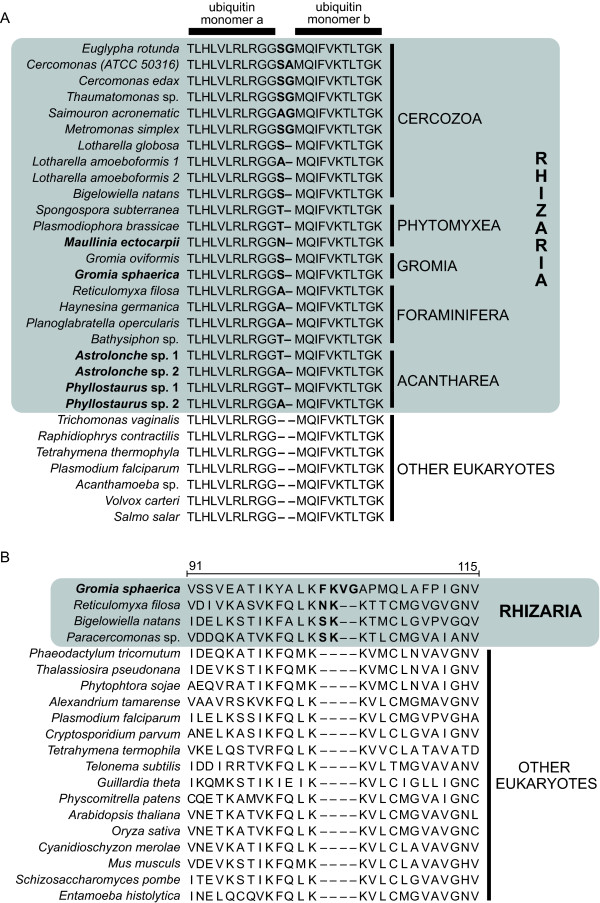
**Specific insertions in Rhizaria**. Rhizarian specific insertions of (**A**) 1-2 residues between monomers in polyubiquitin and (**B**) 2 residues at position 103 in the 60S ribosomal protein L10a. Numbers above the alignment shows the sequence position in the *Mus *protein. Species names in bold indicate new sequences generated in this study.

In addition to the multigene and actin analyses, we screened our newly generated data for the presence of molecular signatures characteristic of Rhizaria. First, polyubiquitin sequences were searched for the 1 or 2 amino acid insertion previously described at the monomer-monomer junction in all Rhizaria except in Radiolaria [17,22]. We found threonine (T) in 4 sequences of *Astrolonche *and one sequence of *Phyllostaurus *and alanine (A) in 6 sequences of *Phyllostaurus *(Figure 8A). The presence of 2 different amino acids in *Phyllostaurus *was surprising, but this is not exceptional as it has already been observed in *Lotharella amoeboformis *(AY099125) where both A and S insertions have been found [17]. A serine (S) was also found in the *Gromia sphaerica *sequence, which was identical to the available polyubiquitin of *Gromia oviformis *(AY571670). In addition, a new polyubiquitin sequence amplified from the phagomyxid *Maullinia ectocarpii *was included.

Interestingly, we identified a new insertion of 2 and 4 amino acids in the 60S ribosomal protein L10a, a characteristic also apparently unique to Rhizaria. A phenylalanine (F), an asparagine (N), and a serine (S) followed by a lysine (K) were inserted at position 104 in *G. sphaerica*, *R. filosa*, *B. natans*, and *Paracercomonas *sp., respectively (Figure 8B). In *G. sphaerica*, the sequence contained 2 additional inserted amino acids, i.e. a valine (V) and a glycine (G). Unfortunately, this gene was not present in the acantharean dataset and several attempts to amplify it by PCR failed. Blast searches against GenBank-nr and dbEST revealed no other known rpl10a gene containing this insertion.

## Discussion

This study provides the first robust evidence for a relationship between Foraminifera and Acantharea, a member of Radiolaria. This result is rather surprising, taking into account the considerable differences in morphology, composition of the skeleton, and lifestyle between these groups. Radiolarians have intracellular celestite (SrSO_4_) (in Acantharea) or siliceous (in Polycystinea) skeleton consisting of strontium sulphate and are holoplanktonic. In contrast, the foraminiferal skeleton (when present) is extracellular, agglutinated or calcareous, and the majority of foraminiferans are benthic. Pseudopodia morphology is also markedly different: radiolarians possess stiff, ray-like pseudopodia called axopodia, while foraminifers are defined by the presence of fine, anastomosing granuloreticulopodia. Still, there are also common cell characteristics shared between these 2 groups, the importance of which must be re-evaluated in view of our data. For example, the network of fine reticulopodia observed in some radiolarians exhibits bidirectional streaming, and is used for capturing prey and locomotion in a similar manner as the foraminiferal granuloreticulopodia [29]. Further studies of proteins involved in pseudopodial formation in both groups are needed to examine these properties at the molecular level. In that respect, it is interesting to note that our acantharean ESTs contained an unusual beta-tubulin strongly resembling the highly diverging type 2 sequences reported in Foraminifera by [33], as well as a less diverging isoform weakly grouping with a new foraminiferan beta-tubulin type (here named "type 3") found in *R. filosa *cDNA library (Additional file 2).

The clustering of Foraminifera and Acantharea observed in our analyses partially confirms the Retaria hypothesis [26]. Although multigene data for the 2 other main groups of Radiolaria, Polycystinea and Taxopodida, are still unavailable, we predict that they will also group with Foraminifera. This relationship is suggested by the phylogenetic position of three fast-evolving sequences of polycystinean actin as sister to foraminiferan actin paralogue 2 [19] as well as by the grouping of Foraminifera with environmental clones assigned to Polycystinea and *Sticholonche *in an analysis of combined SSU and LSU rDNA [25,34]. However, the branching order of these groups was uncertain and Foraminifera may in fact branch within the radiolarian clade, suggesting that Radiolaria (Radiozoa) could be paraphyletic [25,34,35]. The next challenge will be testing whether this surprising pattern arises due to an artifact of LBA, and testing the monophyly of radiolarians.

Further effort is also required to resolve the relationships among the rhizarian groups. Thus far, all phylogenetic studies of this supergroup have recovered Radiolaria alone, or together with Foraminifera as the most basal clade [16]. The latter topology was supported by our LG and WAG-based tree reconstructions (Figure 1), but not by the Bayesian inference with the CAT model (Figure 2). Instead, this method suggested that Retaria are closely related to *Gromia *and Phytomyxea, to the exclusion of Cercozoa. Although this association received only low support with the full dataset, it was strengthen by the experiments with the foraminiferans or the fast-evolving sites removed, as well as by the AU and cross-validation tests. The removal of *R. filosa *was particularly informative in indicating that this species alone could have attracted Retaria at the base of Rhizaria in the "LG" tree, due to its high rate of evolution. Indeed, when it was not included, the topology suggested by the "CAT" model was robustly recovered. Acanthareans, on the other hand, seemed to be less prone to LBA as they consistently branched in a derived position when both foraminiferans were removed. The fast-evolving sites removal analysis also convincingly supported the grouping of Retaria, *Gromia*, and Phytomyxea, and was in agreement with the properties of the CAT model; it has been shown that this model infers homoplasies better than empirical models (such as the LG model used in the RAxML analyses) [36]. Therefore, it might be interpreted that for our complete dataset, the CAT model detected and correctly interpreted more of the saturated positions that were misleading in the RAxML analysis. Within this group, the position of *Gromia *could not be inferred with precision as it branched either as sister to Retaria or Phytomyxea. However, the grouping of *Gromia *with Phytomyxea was recovered only when Acantharea were absent or *R. filosa *was included in the analyses with the "LG" model. Moreover, the better fitted CAT model robustly placed *Gromia *in a sister position to Retaria. Interestingly, the association of Foraminifera, Acantharea, *Gromia *and Phytomyxea has never been described, although SSU and actin trees showed generally unsupported relationships between some but not all lineages [37,38]. In addition, other lineages, such as Haplosporidia or *Filoreta *also belong to this group and will likely be crucial for resolving the internal branching order.

Finally, our study clarifies the question whether acanthareans and polycystines truly lack the rhizarian-specific polyubiquitin insertion, as previously reported [22]. To explain the apparent absence of the insertion in these two groups, it has been proposed that it was lost in radiolarians, or was acquired after their divergence [22]. In our EST data, both acantharean species feature polyubiquitin sequences with the insertion, suggesting that the sequences presented in [22] were not of acantharean origin, but perhaps originated from unidentified symbionts.

## Conclusions

Our multigene analysis elucidates the relationship between two important rhizarian phyla, Foraminifera and Radiolaria (Acantharea), which has been a matter of recent controversy. Because Acantharea do not fully represent the radiolarian diversity and genomic data for other important groups (Polycystinea and Taxopodida) is still missing, we cannot rule out the possibility that Radiolaria are paraphyletic. Nevertheless, our study strongly indicates that a basal position of Radiolaria with respect to the rest of Rhizaria is highly unlikely. Instead, our analysis suggests a novel grouping including Foraminifera, Radiolaria, *Gromia *and Phytomyxea. Within this group, *Gromia *might be most closely related to Foraminifera and Radiolaria, but its specific phylogenetic position will depend on other important lineages such as Haplosporidia or *Filoreta*.

## Methods

### Collecting and isolation of specimens

*G. sphaerica *was collected near Little San Salvador Island in the Bahamas at about 720 m depth (24°34.5'N; 076°00.1'W) and total RNA prepared as described in [28].

Acanthareans were collected during May-June 2008 at the outlet of the Villefranche Bay, Mediterranean sea (43°41'N; 7°18'48E). Plankton samples were taken using a plankton net (mesh diameter 20 μm) drawn vertically from the depth of 200 to 0 m. Concentrated samples were immediately brought to the lab and processed. Living acanthareans were picked from the plankton with needles, washed with filtered seawater and placed in RNAlater solution (Ambion). The solution was allowed to penetrate into the cells for 24 hours at 4°C, after which the samples were kept frozen until further processing. In total about 300 cells of *Phyllostaurus *and 50 cells of *Astrolonche *were collected and used for library preparation.

The 2 phytomyxean samples were prepared from *in vitro *grown callus consisting of *S. subterranea *infected *Solanum tuberosum *cells and *P. brassicae *infected *Brassica rapa *cells, respectively. Full details of the generation, growth and characterization of these callus lines will be detailed elsewhere (Bulman et al. submitted). Briefly, sections of *S. subterranea *or *P. brassicae *root galls were surface sterilized and placed on MS media. Segments of white/green multiplying cells were transferred to new media as they proliferated. Fresh green callus cells were harvested and transferred into RNAlater (Qiagen).

### Preparation of cDNA libraries for 454 sequencing

The *G. sphaerica *cDNA library was prepared from approximately half of an individual, as described in [39], with minor modifications (described below) that ensured enrichment of the data with protein-coding sequences. Briefly, the methodology involves cDNA synthesis and amplification using SMARTer cDNA synthesis kit (TaKaRa BIO/Clontech, Mountain View, CA), normalization using Trimmer kit (Evrogen, Moscow, Russian Federation), fragmentation by sonication, end-polishing, ligation of adaptors, and amplification of the 454-ready sample. The design of the adaptors ensures that the sequencing proceeds only from the cDNA breaks introduced by sonication, rather than from original termini, which helps to reduce the amount of adaptor-derived sequences in the resulting data.

For acanthareans, we originally prepared the libraries from 100 *Phyllostaurus *and 20 *Astrolonche *cells using the same protocol without normalization, but after Sanger-sequencing 24 randomly picked clones per species we found that the libraries consisted predominantly of non-coding sequences, most likely representing 3'-UTRs of the original transcripts. To enrich our libraries with the coding regions, we amplified the SMARTer kit-synthesied cDNA with a long primer (5'-AGTGGACTATCCATGAACGCAAAGCAGTGGTATCAACGCAGAGT-3') at a concentration of 0.1 μM, instead of using the one included in the kit, which resulted in preferential amplification of the longest cDNA fragments due to mild PCR-suppression effect [40]. Moreover, after fragmentation and adaptor ligation, only the 5'-ends of the original cDNAs were amplified and subjected to sequencing, by using the primer annealing to the ligated sequencing adaptor [39] and the primer matching to the unique sequence of the template-switch oligonucleotide used during the cDNA synthesis (5'-GCCTCCCTCGCGCCATCAGCCGCGCAGGTACGTATCAACGCAGAGTACGCGG-3'). The libraries were sequenced using 454 GS-FLX. The latest version of the cDNA preparation protocol, adapted for the latest 454 version (Titanium), is available on Matzlab website [41].

For the 2 phytomyxean species, cDNA libraries were constructed by Vertis Biotechnology AG (Germany) according to their Random-Primed (RPD) cDNA protocol. Frozen cells were ground under liquid nitrogen and total RNA isolated from the cell powder using the mirVana RNA isolation kit (Ambion). Poly(A)+ RNA was prepared from total RNA. First-strand cDNA synthesis was primed with an N6 randomized primer and second-strand cDNA was synthesized according to the classical Gubler-Hoffman protocol [42]. Double stranded DNA (dsDNA) was blunted and 454 adapters A and B ligated at the 5' and 3' ends. dsDNA carrying both adapter A and adapter B attached to its ends was selected and amplified with PCR using a proof reading enzyme (24 cycles). For 454 sequencing the cDNA in the size range of 250 - 600 bp was eluted from a preparative agarose gel.

### Contig assembly and sequence alignment

All newly generated reads were assembled into contigs using the Newbler assembler with default parameters, generating the following number of contigs larger than 100 bp: *G. sphaerica*, 24,433; *Astrolonche *sp., 6426; *Phyllostaurus *sp., 5056; *P. brassicae*, 27,333; *S. subterranea*, 14,531. To filter plant sequences out of the phytomyxean datasets, the phytomyxid-callus contigs were compared to plant cDNA sequences using the BLAT tool [43] and those with very high similarity were discarded (e-value threshold < 1e-50). The 27,333 *P. brassicae *contigs (containing *B. rapa*) were compared against 2,529,141 Brassicaceae ESTs (NCBI as of 25 March 2009, http://www.ncbi.nlm.nih.gov/) and resulted in 24,166 contigs showing nearly identical hits. The 14,531 *S. subterranea *contigs (containing *S. tuberosum*) were compared to 1,000,784 Solanaceae ESTs (NCBI as of 25 March 2009, http://www.ncbi.nlm.nih.gov/), giving 8,282 contigs with a nearly identical hit. Manual inspection (using blastn) of a subset of the filtered sequences confirmed that these were indeed likely to originate from the plant host cells. The remaining 3,167 *P. brassicae *contigs with low or no hit against the Brassicaceae ESTs and the 6,249 *S. subterranea *contigs with low or no hit against the Solanaceae ESTs were used in subsequent screenings.

Blast searches against databases containing the 5 species above and taxa downloaded from GenBank (http://www.ncbi.nlm.nih.gov/) and JGI (http://genome.jgi-psf.org/) were performed to retrieve the genes of interest (see Table S1 for the list of species). These genes corresponded in part to genes that we used in previous phylogenomic studies [3,4,7], but also to 55 genes representing additional members of large protein families that were not previously included in our alignment, such as more minichromosome maintenance proteins (MCM) or more proteasome subunits, but also several new ribosomal proteins and proteins for which a broad sampling was available (see Additional file 3 for a list of the newly added genes). In total, 202 single-gene alignments were constructed, automatically aligned with Mafft [44], using Gblocks [45] to remove ambiguously aligned positions (with half of the gapped positions allowed, the minimum number of sequences for a conserved and a flank position set to 50% of the number of taxa plus one, the maximum of contiguous non-conserved positions set to 12, and the minimum length of a block set to 5) and followed by manual adjustment when needed with BioEdit [46]. The orthology and possible contamination in each gene was tested by ML reconstructions with 100 bootstrap replicates using RAxML 7.2.2 (LG substitution matrix) [47], and visual check of the resulting individual trees. Out of the complete set of 202 genes, 23 showed evidence for deep paralogy and were therefore discarded. In addition, we also excluded 12 extra genes as they did not contain any species of Rhizaria. Our final dataset contained 167 genes, which represented 36,735 amino acid positions after concatenation, and 39 species belonging to Rhizaria, stramenopiles, alveolates and green plants (outgroup). The single-gene and concatenated alignments are available at http://www.fabienburki.com. A table listing all genes and a detailed view of the missing data repartition for each taxa can be found in Additional file 1. The 167 trees constructed from the final selection of genes are available in Additional file 4. Since many of the Phytomyxea contigs were short due to the relatively small number of contigs that were obtained from the phytomyxid parasites, 35 genes were chosen as targets for further sequence acquisition. PCR primers were designed based on the short contigs and longer DNA sequences were obtained by RT-PCR or 3'RACE using the callus RNA as template (as in [48]). Scafos 1.2.5 [49] was used for performing the concatenation process of the single-genes.

The actin and beta-tubulin alignments were built by retrieving from GenBank sequences belonging to all rhizarian (actin) or eukaryotic (beta-tubulin) groups, and adding to them the sequences identified in the datasets generated in this study. 72 rhizarian and 6 stramenopiles (outgroup) sequences were included in the actin tree; 119 eukaryotes were analyzed for the beta-tubulin tree. We also searched our datasets for the 1 or 2 amino acids insertion at the monomer-monomer junctions carried in most rhizarian species. Finally, the construction of the single-gene alignments revealed a rhizarian specific insertion of 2 amino acids in the 60S ribosomal protain L10a.

### Phylogenetic analyses

ML analyses were performed using RAxML 7.2.2 [47] in combination with the LG amino acid replacement matrix [50]. The best ML tree was determined with the PROTGAMMA + F implementation in multiple inferences using 10 randomized parsimony starting trees. Statistical support was evaluated with 100 bootstrap replicates. Bayesian analysis using the WAG + G + F model (4 gamma categories) was done with the parallel version of MrBayes 3.1.2 [51]. The inference consisted of 1,000,000 generations with sampling every 100 generations, starting from a random starting tree and using 4 Metropolis-coupled Markov Chain Monte Carlo (MCMCMC). 2 separate runs were performed to confirm the convergence of the chains. The average standard deviation of split frequencies was used to assess the convergence of the 2 runs. Bayesian posterior probabilities were calculated from the majority rule consensus of the tree sampled after the initial burnin period as determined by checking the convergence of likelihood values across MCMCMC generations (corresponding to 50'000 generations). PhyloBayes 3.1 [52] was run using the site-heterogeneous mixture CAT model with the rates-across-sites heterogeneity handled by a Dirichlet process (ratecat). 2 independent Markov chains with a total length of 19'000 cycles were performed, discarding the first 2'000 points as burnin, and calculating the posterior consensus on the remaining trees. Convergence between the 2 chains was ascertained by examining the difference in frequency for all their bipartitions (< 0.1 in all analyses). Bootstrap CAT proportions were obtained after 5000 cycles with a conservative burnin of 1000 on 100 pseudo-replicates generated with Seqboot (Phylip package [53]). Manual verification of 10 replicates showed that the burnin is generally between 500-700 cycles. For each replicate, trees were collected after the initial burnin period and a consensus tree was computed by readpb (PhyloBayes package). Consense (Phylip package [53]) was then used to calculate the bootstrap support based on these 100 consensus trees. Due to limited size of single-genes for parameter estimation under non-parametric models such as CAT, the PhyloBayes-based actin and beta-tubulin phylogenies were ran under the LG model.

The site-removal analysis was performed using PAML [54] to identify the fast-evolving sites, as implemented in the AIR package [55]. Because the topology chosen to estimate the site-wise rates strongly influences the results [32], rates were calculated for the 105 possible different topologies describing the evolutionary relationships of the 5 rhizarian lineages, and sorted according to the mean of the rates estimated on all topologies. 5% to 90% (10% intervals between 10% and 50%, 5% intervals between 55% and 90%) of the fastest evolving sites were then removed (percentage of the total rate distribution), and bootstrapped ML analyses were run with each of these 14 shorter alignments. PhyloSort [56] was used to search the pseudo-replicate trees for the relationships of interest. Phylobayes analyses with the CAT model were also done on each reduced alignment.

The statistical model comparison was done using the cross-validation (CV) method available in PhyloBayes 3.1 [52]. A learning and a test sets were generated by randomly splitting (no replacement) the original alignment into 10 replicates made of 90% and 10% of the original sites, respectively. Each of the learning and test alignments amounted to 33,062 and 3673 positions, respectively. A MCMC run was performed for each replicate under a fixed topology (either the "CAT" or "LG" tree) for a total of 5000 cycles ("CAT") or 1500 cycles ("LG"). Due to technical reasons associated with this test in PhyloBayes, a discrete gamma distribution with 4 categories was used for modelling the rate heterogeneity across site (i.e. dgam instead of ratecat). This slightly different model should not affect the conclusions given the big difference between CAT and LG. The lower number of cycles under "LG" was due to a much greater computational time per cycle as compared to when the "CAT" model was used. The first 500 and 150 points were discarded as burnin for the "CAT" and "LG" runs, respectively, and the remaining points used to compute the cross-validation log-likelihood.

Topology comparisons were conducted using the approximately unbiased (AU) test [30]. For each tested tree, site likelihoods were calculated using RAxML 7.2.2 with the LG model and the AU test was performed using CONSEL [57].

## Authors' contributions

FB and JP conceived the project. AK collected the acanthareans. MVM and GVA collected *G. sphaerica *and prepared the cDNA libraries for *G. sphaerica *and the acanthareans. SB isolated the plasmodiophorids. MF analyzed the plasmodiophorid ESTs. FB prepared the alignments and performed the phylogenetic analyses. FB, AK, MVM, GVA, SB, PJK and JP contributed to writing the manuscript and approved its final version.

## Accession numbers

454 reads generated in this study for *G. sphaerica*, *Astrolonche *sp. and *Phylostaurus *sp. were deposited in GenBank under the study accession SRP004044.1, and for *P. brassicae *and *S. subterranea *under the study accession SRP003604.2. The RACE products for *P. brassicae *and *S. subterranea *were deposited in GenBank under the accession HO772678-HO772709. The new *Maullinia ectocarpi *polyubiquitin sequences were deposited in Genbank under the accession HQ366774-HQ366778.

## Supplementary Material

Additional file 1 **Percentage of missing data**. Gene list with percentage of missing data indicated for each OTU Click here for file

Additional file 2 **ML phylogeny of eukaryotes based on beta-tubulin**. Numbers at nodes represent the bootstrap values obtained with RAxML ("LG" model) and the posterior probabilities obtained with PhyloBayes ("LG" model). For clarity, only the values for the nodes of interest for this study are shown (most of the among groups relationships were unsupported). The scale bar represents the estimated number of amino acid substitutions per site. Click here for file

Additional file 3 **New genes used in this study**. Table listing new genes used in this study (i.e. not included in Burki et al. 2007, 2008, 2009) Click here for file

Additional file 4 **Single gene trees**. 167 trees inferred from the single-gene alignments included in the supermatrix using RAxML 7.2.2, 100 bootstrap replicates, LG + gamma + F Click here for file
